# HPV-Based Self-Sampling in Cervical Cancer Screening: An Updated Review of the Current Evidence in the Literature

**DOI:** 10.3390/cancers15061669

**Published:** 2023-03-08

**Authors:** Nikoletta Daponte, George Valasoulis, Georgios Michail, Ioulia Magaliou, Athina-Ioanna Daponte, Antonios Garas, Ioanna Grivea, Dimitrios P. Bogdanos, Alexandros Daponte

**Affiliations:** 1 Department of Obstetrics & Gynaecology, Faculty of Medicine, School of Health Sciences, University of Thessaly, 41500 Larisa, Greece; 2 Hellenic National Public Health Organization-ECDC, Marousi, 15123 Athens, Greece; 3 Department of Midwifery, School of Health Sciences, University of Western Macedonia, 50100 Kozani, Greece; 4 Department of Obstetrics & Gynaecology, Faculty of Medicine, School of Health Sciences, University of Patras, 26504 Patras, Greece; 5 Second Department of Dermatology-Venereology, Aristotle University School of Medicine, 54124 Thessaloniki, Greece; 6 Department of Paediatrics, Faculty of Medicine, School of Health Sciences, University of Thessaly, 41500 Larisa, Greece; 7 Department of Rheumatology and Clinical Immunology, Faculty of Medicine, School of Health Sciences, University of Thessaly, 41500 Larisa, Greece

**Keywords:** human papillomavirus, HPV, self-sampling, cervical cancer, screening, secondary prevention, HPV-biomarker, HPV DNA, HPV mRNA

## Abstract

**Simple Summary:**

Dynamics in global health adaptations to the SARS-CoV-2 pandemic culminated in a unique momentum to reform cervical screening by reinforcing self-sampling implementation. Aside from catch-up screening, self-sampling has established a continuing role in increasing cervical cancer screening uptake and scaling coverage globally. Thus, self-sampling has evolved as a key pillar of the ambitious global strategy of the WHO to eliminate cervical cancer. By providing opportunities and screening alternatives for women and by lowering the barriers to screening participation, self-sampling may ultimately reduce national, racial and social health disparities in cervical cancer rates.

**Abstract:**

Identifying and reaching women at higher risk for cervical cancer is all-important for achieving the ambitious endpoints set in 2020 by the WHO for global cervical cancer control by 2030. HPV-based (vaginal) self-sampling (SS) represents a cost-effective screening strategy, which has been successfully implemented during the last decade both in affluent and constrained settings. Among other advantages, SS strategies offer convenience, diminished costs, flexibility to obtain a sample in the office or home, avoiding a pelvic exam and uncomfortable appointment with a healthcare professional, as well as social and cultural acceptability. SS implementation has been globally boosted during the COVID-19 pandemic. In pragmatic terms, social distancing, local lockdowns, discontinuation of clinics and reallocation of human and financial resources challenged established clinician-based screening; self-collection strategies apparently surpassed most obstacles, representing a viable and flexible alternative. With time, sufficient reassuring data has accumulated regarding specially designed SS devices, aspects of sample preparation, transport and storage and, importantly, optimization of validated PCR-based HPV testing platforms for self-collected specimens. Suboptimal rates of clinical follow-up post-SS screening, as well as overtreatment with reliance solely on molecular assays, have both been documented and remain concerning. Therefore, effective strategies are still required to ensure linkage to follow-up testing and management following positive SS results by trained health professionals with knowledge of HPV biology and management algorithms. Because of the prolonged SS screening intervals, implementation data are limited regarding subsequent screening rounds of SS-screened individuals; however, these are accumulating gradually. With further refinement of assays and validation of novel biomarkers in self-collected samples, there is a clear potential for increasing SS accuracy and PPV. The potential differentiation of self-collection protocols for vaccinated versus non-vaccinated individuals also represents an open issue. In conclusion, HPV-based self-collection techniques can effectively address limited uptake alongside other conventional cervical screening drawbacks; however, assays, logistics and infrastructures need further optimization to increase the efficacy, effectiveness and cost-effectiveness of SS approaches.

## 1. Introduction

The interim from HPV infection to cervical cancer will usually take 10–20 years or longer, thus leaving a great opportunity for screening and early detection. Established organized cervical cancer screening (CCS) programs, with central audits and strategies enabling maximum participation, represent a prerequisite and the basis before a successful primary HPV screening can be implemented in a health system. 

The advantages of organized cervical screening over the opportunistic approach, mainly in cost-effectiveness terms, have been long established. Linking organized screening with central registries maximizes potential benefits, especially when the number of certified participating laboratories processing cervical specimens is limited, quality assured and audited by a supervising authority (agency, prefecture, central government etc.) [1].

In the previous decades, HPV-based screening has consolidated its superiority over cytology in mass cervical screening. Accumulating data and several meta-analyses have illustrated improved negative predictive value and sensitivity for HPV molecular screening compared with cytology-based strategies; furthermore, the safe extension of screening intervals to five or more years between screening rounds contributes to superior logistics and cost-effectiveness [2,3,4,5]. Initial interim guidelines have been globally dismissed with accruing evidence of co-testings’ diminished cost-effectiveness at the population level, especially in the US, which favored co-testing (HPV test and simultaneous cytology) as a failsafe safeguard [6].

For women unwilling to engage in clinician-obtained cervical screening, both urine and vaginal self-sampling (SS) represent valid alternative approaches despite the reported marginally inferior accuracy of urine when compared with vaginal SS [7]. As we have already recently published an updated review on urine SS, in this manuscript, we will focus on emerging aspects of vaginal SS during the prolonged SARS-CoV-2 pandemic [8].

## 2. Aspects of Vaginal Self-Sampling Implementation

Wide population coverage, as well as screening uptake, is paramount for successful cervical screening implementation. Broadening the target population and enhancing participation rates are crucial, both in affluent and more constrained settings. A recent meta-analysis of one-hundred-fifty-four HPV SS studies globally identified, involving 482,271 women, concluded that SS procedures nearly doubled the probability (RR: 1.8; 95% CI: 1.7–2.0) of CCS uptake when compared with clinician-collected samples [9].

In May 2020, shortly after the outbreak of the SARS-CoV-2 pandemic, the WHO called on all global stakeholders involved in the prevention of CC to take serious action to eliminate this disease as a population-wide problem. In this regard, the WHO has determined three complementary mid-term objectives to be reached by the year 2030, aiming to enhance the chances of achieving the aforementioned goal. This minimum set of targets, related to vaccination coverage, implementation of accurate screening tests and adequate treatment, is widely known as the “90-70-90” goals [10,11]. The reallocation of resources due to the COVID-19 pandemic and the WHO cervical cancer elimination targets necessitate the introduction and wider use of SS in cervical screening.

Besides economic and reimbursement obstacles, which are universal CCS barriers in ethnically and culturally diverse societies, certain population clusters are more likely to abstain from established screening pathways and offerings; however, they might engage in SS screening. For several individuals or groups, numerous potential barriers to participating in the conventional procedure of clinician-obtained cervical smears have been identified. Arguably, the major barrier for women across many cultures has been the requirement to undergo a speculum examination [12]. Among others, elevated costs or non-reimbursement, non-convenient clinic hours, provider-related issues (inability to locate, poor communication, etc.), difficulties with transportation, feelings of embarrassment or pain [13], religious or cultural beliefs, male healthcare providers, fear of the procedure itself or its results, history of sexual trauma or abuse and lack of time or willingness to prioritize preventative health (perceived needs) have also been identified [14].

The high acceptability among women of vaginal SS as a screening strategy has been repeatedly demonstrated in various publications [15]. Most cited advantages include skipping the pelvic exam, convenience of completing the procedure, privacy, absence of embarrassment (dignity) and comfort. Some minor disadvantages have been reported, namely pain and physical discomfort, anxiety, uncertainty regarding obtaining sufficient material for testing and embarrassment in touching themselves [16]. The adherence to screening programs in some areas of the world remains very low due to all the above-mentioned reasons, and HPV testing through SS has gained attention for its potential to increase screening participation. SS can be utilized to scale up population screening coverage, with improved attendance among under-screened and hard-to-reach women [17]. Furthermore, no adverse events or social harms have been identified for SS practices [18].

Women mostly appreciate several benefits of vaginal SS, such as ease and convenience of use, privacy and value of their direct participation and involvement in healthcare [16]. For women, it appears that the most appealing features of SS are cost (nil or minimal), convenience (home-based) and less anticipated discomfort than a Pap smear [7,19].

### 2.1. Which Self-Sampling Device?

Numerous devices have been assessed in vaginal SS studies in a variety of settings [12]. Most are readily available commercially; despite not currently being FDA-approved, several have been validated in the framework of the VALHUDES protocol. Per principle, all devices aim to harvest exfoliated cells from the cervicovaginal canal for subsequent HPV DNA detection. The four main forms consist of cervicovaginal brushes, vaginal swabs, lavage devices, as well as tampon-like/patch devices [20]. One meta-analysis of Arbyn et al. illustrated that brush- and swab-based devices were a little more sensitive (98%, 95% CI 0.93–1.03) than lavage-based devices (95%, 95% CI 0.87–1.04) [21]. The experience so far is that women might have a preference for smaller devices (brush- or swab-based), which are colorful in appearance. 

Initial skepticism towards SS was mainly attributable to the fear of obtaining a suboptimal sample and questionable diagnostic performance [22]. However, this wide range of differently designed sampling devices appears to produce satisfactory results, which is clearly reflected in the 2018 Arbyn meta-analysis [12]. Equally, no major differences have been documented between sampling devices in terms of hrHPV-positivity or sample-inadequacy rates [21,23]. Thus, several contributing factors might dictate the choice of the device within a SS program, namely an affordable cost per unit, stability over time in extreme temperatures, flexibility for high volume throughput, etc. [12]. The choice of HPV SS device type offerings may also affect women’s acceptability of SS; in this context, brush-based devices have been previously linked with enhanced participation [7]. 

In the Di Gennaro et al. meta-analysis, a higher relative uptake was shown for brushes (RR: 1.6; 95% CI: 1.5–1.7) and swabs (RR: 2.5; 95% CI: 1.9–3.1) over clinician-collected samples (RR: 1.8; 95% CI: 1.7–2.0), the authors concluded that since no significant difference in acceptability and preference of device type was demonstrated among women and swabs and brushes exhibited a potential stronger effect in improving SS performance, these devices could be therefore adopted [9].

### 2.2. Which Self-Samplers’ Distribution Strategy?

Because of the crucial impact on uptake and response rates, optimizing alternative pathways for attractive SS delivery has gathered much attention. Established organized screening procedures usually rely on mailed reminder letters calling for the collection of clinician-obtained samples. The evolution of social media and multimedia platforms has unprecedentedly facilitated the delivery of cervical screening worldwide. Globally, the main approaches for SS delivery are either an invitation to order the HPV-SS kit (“opt-in”) or a directly mailed HPV-SS kit (“send-to-all” / “opt-out”), which is more expensive. Rebolj et al. commented that despite the “opt-out” approach usually resulting in higher uptake, the “opt-in” also represents an effective alternative, particularly when supported with suitable communication platforms that simplify the kit-ordering process [24]. In that line of thought, an excellent paradigm of successful SS implementation comes from Malaysia, where the start-up “ROSE” (Removing Obstacles to cervical Screening) primary HPV-based cervical screening program successfully integrated current advances in SS, with HPV screening and innovative digital platforms to invite women to participate. In this environment, a vital connection to care is facilitated through the use of digital registries [25].

Rebolj M. et al. further comment on the inherent flexibility of SS in approaching under-screened women and quote on innovative SS delivery pathways, such as the opportunistic offer of an SS kit in primary care to women attending for unrelated reasons, organizing the distribution of SS kits through a network of local pharmacies, recruitment through home visits by health care workers or via community campaigns and other outreach activities [24].

An updated systematic review and meta-analysis on the effectiveness of strategies to increase participation in SS initiatives are described in detail in the paper by Costa et al. [26]. Besides the aforementioned “mail-to-all” and “opt-in” strategies, the authors evaluate the “community mobilization and outreach” scenario, as well as the “direct offer at a healthcare service” option. In this meta-analysis, the highest participation was achieved with strategies involving a face-to-face invitation, potentially because of an increase in the women’s confidence to perform the self-collection. The authors documented the highest absolute gain in reaching under-screened populations when SS kits were offered through community mobilization and outreach. The “mail-to-all” strategy was considered more effective in generating uptake than the “opt-in” and could be considered in high-income countries; the per-protocol participation in the “opt-in” scenario was overall not significantly more effective than with conventional invitations. Remarkably, SS offers as a healthcare service also represent a promising strategy to improve screening participation rates. Since no universal patterns apply, pilot implementation studies should be conducted to identify and tailor the best strategy to each population while introducing vaginal SS, carefully evaluating its viability and appropriateness in the specific local context before a generalized roll-out [26,27,28].

In the Di Gennaro et al. meta-analysis, the “opt-out” and the “door-to-door” did not statistically significantly differ (*p* = 1.177) in improving SS uptake and, therefore, should be recommended, dependent on the available resources.

### 2.3. Which HPV Tests?

Currently, HPV assays based on polymerase chain reaction (PCR) are mostly implemented in SS; this is predominantly attributed to the lower specificity of signal amplification tests in self-samples [29,30]. Based on the suboptimal sensitivity of mRNA assays for detecting underlying CIN2+ in self-collected samples compared with clinician-collected samples, DNA assays are preferred [31].

### 2.4. Validated Assays—Sample Preparation and Pre-Analytic Considerations

The clinical validation of an assay represents a minimum requirement for the adoption of a molecular platform in a SS CCS program; assays can be scrutinized within the two major platforms for assessing clinical sensitivity and specificity, namely the Meijer Criteria or the VALGENT framework [12]. Globally, Roche’s Cobas^TM^ platforms are currently mostly utilized. Despite several assays performing well in vaginal SS, as evidenced in the carefully selected populations of published studies, none have been validated with an FDA approval or CE mark so far [12].

Several steps during the sample preparation of SS specimens in centrally accredited laboratories are critical for quality assurance in SS strategies. This especially applies when specimens arrive at the laboratory in a “dry sample” form, to be later re-suspended in liquid media [12]. Manufacturers are urged to adapt and optimize their platform’s standard protocols for SS implementation; this will simplify pathology laboratory accreditation for self-collection and also enhance daily output [8,12]. With the anticipated growing availability of self-collection, laboratories’ caseload will predictably rise exponentially, requiring investments in pre-analytic automation, both for coping with the increased workflow and for the reduction in potential inter-operator errors [8,12].

### 2.5. Time-Lapse of Global SS Implementation–Resolved Obstacles and Emerging Challenges

A decade ago, the available HPV molecular platforms had not been optimized yet for use in SS material and therefore performed inferiorly, both analytically and clinically. In this era, an earlier paper by Rozemeijer et al. underscored that SS should be endorsed with vigilance and that the advantages of office-based screening needed to be emphasized to prevent the switching of regularly screened individuals to SS. The authors illustrated that under a constellation of assumptions, the QALYs gained by attracting non-attendees into SS could possibly be annulled by the QALYs lost by the switching of regular attendees to SS [32]. Driven by this skepticism, the recent paper by Rebolj et al. attempts a critical appraisal of current SS implementation [24]. The authors focus on pragmatic data derived from early SS adopters instead of mathematical modeling or projections in the studied populations. Concerns expressed are related to diminished program performance in the theoretical co-occurrence where low SS uptake from under-screened women is combined with switching clinician sampling to self-collection among well-screened women, together with suboptimal CIN2+ detection rates in SS compared to clinician-obtained sampling. The authors quantify these assumptions by providing subsets of combinations for these three parameters for which the system’s performance could be potentially threatened in a critical manner. They substantiate their standpoint by opposing real-world dysfunctions, which emerged during SS implementation in the Netherlands and Australia.

The Netherlands was the first European country to introduce HPV primary screening in January 2017; the new program started only after a preparation phase of more than 4 years [1]. The Dutch screening program was the first to offer SS (in an “opt-in” mode) for women who would otherwise not attend; non-responders received a reminder letter four months following the first invitation with guidance on how to request the SS kit. However, during the first two years of SS availability, this was not widely promoted centrally. There is evidence that during these first two years of primary hrHPV screening implementation in the Netherlands, women who used SS differed from women who chose to be screened by the GP in terms of screening history and socio-demographic characteristics. In the Netherlands, SS could target under-screened women as a more suitable, user-friendly primary screening tool [33].

In Australia, since December 2017, the national cervical screening program switched to five-yearly primary HPV-based testing to begin at the age of 25. Until July 2022, the SS option was restricted to under- or never-screened women aged 30 years old and older or those who refused a speculum exam [14]. SS implementation faced several serious difficulties, being hampered both by unclear communication and lack of promotion to providers, as well as decreased availability of accredited laboratories processing self-collected samples. The main stakeholders, primary care providers tasked to communicate and offer SS, were poorly guided regarding the pathology processes, availability, clinical management pathways for self-collection and participant eligibility. Furthermore, a regulatory delay in developing an approved protocol regarding laboratory processing of self-collected swabs meant that initially, only one nationally accredited laboratory could process samples, resulting in lost opportunities and misinformation regarding the pathway’s availability [14]. In the Australian context, Zammit et al. identified several core values which they consider most determining for a successful SS implementation outcome, namely: appropriateness, feasibility, fidelity, penetration, implementation cost and sustainability [14].

Nonetheless, the paper of Rebolj et al. provides insight by proposing several optimizations on SS implementation: Efforts should address already-identified caveats, focusing on (i) improving the uptake among under-screened women, (ii) improving SS sensitivity in CIN2+ detection, (iii) addressing the low compliance with triage testing on the detection of CIN2+, (iv) improving the triage pathway and (v) emerging issues with collection methods [24].

From a diametrically opposite standpoint, the recent publication of Smith et al. suggests a potential routine SS offering as an equivalent alternative within organized CCS [34]. In the Australian context of high vaccination coverage and induced HPV herd immunity, the authors used a well-established model of HPV transmission, natural history, vaccination, cervical screening, and treatment of precancer and cancer. They estimated that for unvaccinated cohorts, the health benefits of increased participation from SS would be outweighed by the worst-case (2%) loss of relative test sensitivity, even for a marginally improved (15%) additional uptake. For vaccinated cohorts, population-wide SS could be marginally (0.2–1.0%) less effective at a low 15% additional uptake but 6.2% to 12.4% more effective at 50% additional uptake. The authors consider that even under pessimistic assumptions, any potential loss in test sensitivity from self-collection is likely outweighed by improved program effectiveness resulting from feasible levels of increased uptake, concluding that SS should be encouraged and offered more widely, potentially as an equal choice for women [34]. Despite cytology representing the commonest triage in clinician-obtained HPV-based cervical screening, morphological analysis performs poorly in self-samples, precluding a role for cytology as a candidate triage test. During the previous decade, molecular research has illustrated that DNA methylation markers and classifiers incorporating methylation gene panels are applicable to self-samples, achieving satisfactory diagnostic performance and having the potential to reduce the risk for undetected cervical cancers and advanced CIN2/3 [35]. Conversely, women with negative DNA methylation tests would probably have low short-term cancer progression risk, indicating that immediate colposcopy referral is unnecessary [35]. While refinement of assays, techniques and laboratory protocols remains an open issue, direct molecular triage on self-collected specimens could optimize the screening program, especially for non-responders, eliminating the need for an additional upfront physician-obtained triage testing [36]. This represents a pragmatic transition to a full molecular self-screening approach in cervical screening programs [35]. Studies assessing the performance of dual immunostaining of p16INK4a and Ki-67 proteins in VSS have not illustrated equally promising results as in clinician-collected liquid-based cytological (LBC) samples [37,38,39].

Currently, cervical surveillance systems are globally facing new challenges as vaccinated cohorts enter the screening age. Withstanding adaptations are the subject of projections and mathematic modeling; however, the lower CC background risk will reflect on the cost-effectiveness of the screening approaches, be they a clinician or SS. As outlined before, structural changes, enhanced screening coverage together with targeting seldom screened individuals and further refinement of techniques are necessary to mitigate the potential loss in the harvest of cervical precancer cases [32]. Under the new paradigm, cytology, which performs poorly in SS, is currently reserved for the triage of individuals testing positive in HPV-based screening. However, adherence to triage in HPV-positive women, irrespectively how the first sample was taken, is a major challenge because the two-step triage strategy is characterized by a degree of dropout at follow-up (f-u) [1]. The ideal management and follow-up of women found HPV-positive in SS is an open field; cytology, partial genotyping as well as colposcopy all represent valid triage strategies. This triage is a difficult challenge because these women, often hard to reach, must be referred to a clinician for cervical cytological sampling [1]. The 2018 Arbyn et al. meta-analysis has clearly illustrated that in several studies, the adherence to f-u requirements was statistically significantly lower in women who tested positive for hrHPV in the SS arm versus women in the control arm (conventional, clinician-provided screening) [21]. More specifically, the results showed that hrHPV assays (based on signal amplification) have a pooled absolute sensitivity for CIN2+ substantially lower in self samples (77%, 95% CI 69–82%) compared with clinician samples (93%, 95% CI 89–96%), while for both self-samples and clinician collected samples the pooled absolute specificity for CIN2+ was 84% (95% CI 77–88%) in self samples and 86% (95% CI 81–90%) in clinician collected samples. The authors concluded that the pooled absolute sensitivity and specificity for both self-samples and clinician-collected samples of hrHPV assays for CIN2+ detection were 96% and 79%, respectively, based on polymerase chain reactions (PCR) [21].

Other authors also stress that the key challenge in implementing SS in any setting is ensuring f-u and treatment for women with abnormal tests, as well as assuring adequate training for supervision and audit of these services [7,18]. By identifying the generally low rate of clinical f-u after a positive SS screening result as a cause for concern, the meta-analysis of Yeh et al. also underscores that strategies for improving linkage to treatment after positive results are urgently needed [18]. Inferior rates of f-u care in low-resource settings than in high-resource ones following an HPV(+)ve SS result has been documented [7,18]. Adequate f-u rates might be influenced by triage policy: studies with direct patient referral exhibit higher attendance at f-u care than studies with a triage policy [21]. In low-resource settings, f-u rates could be optimized by engaging community health workers, ensuring rapid availability of test results and offering mobile community treatment. In high-resource settings, f-u rates could be further improved through pre-booked appointments, personal contacts with non-attenders, prescheduled reminders or reminder letters to HPV-positive women as well as direct contact with a physician [7].

### 2.6. Cost-Effectiveness Considerations

Optimizing the cost-effectiveness of SS strategies, predominantly by streamlined screening protocols, is a prerequisite for successful SS adoption. In SS implementation, the cost-effectiveness profile is basically determined by the socio-demographic characteristics of the target population, intervals between consecutive tests and the pre-selected HPV molecular platform and triage strategy [11].

Irrespective of the selected approach for SS kits distribution, the most prominent reductions in monetary costs associated with SS can be potentially traced to the reduction in unnecessary office-based examinations for women testing HPV-negative, potential decreases in excess colposcopy referrals and subsequent treatments [7]. Another important aspect is discouraging over-screening, or double-screening practices (SS in parallel with in-office visits) [7]. Especially for Low- and Middle-Income Countries (LMICs), enhanced SS cost-effectiveness could be achieved by shifting cancer screening to community-based models, as well as the integration of CC screening with HIV monitoring [7]. 

Both the prevalence of hrHPV and the burden of cervical precancer among HIV patients are alarming, especially in LMIC countries with limited infrastructures [40,41]. With similar laboratory performance of self-sampling procedures to clinician-performed sampling, high participation and reported satisfaction rates for the individuals, there is clear potential for increasing overall cervical cancer screening uptake with VSS in this population and maximizing efficiency and cost-effectiveness, essentially implementing the same networks and infrastructures, with comparatively lower additional costs. This gains additional importance, given the contribution of underserved patients living with HIV in the global burden of cervical precancer and cancer morbidity and mortality [42,43].

In addition to the cost-effectiveness perspective, every screening program requires the careful balancing of benefits and harms [44]. Offering SS to non-attending individuals who are overdue for their screening appointment is seemingly the obvious alternative. However, the assessment of the cost-effectiveness of switching from clinician collected to self-collected HPV testing in cervical screening is inherently complex and highly individualized. Several contributing factors need consideration, among others the setting (affluent or less so), vaccination coverage and herd immunity, as well as the selected triage strategy [45]. Despite limited data, for certain populations switching might be cost-effective under certain simulations [46].

**Figure 1:**
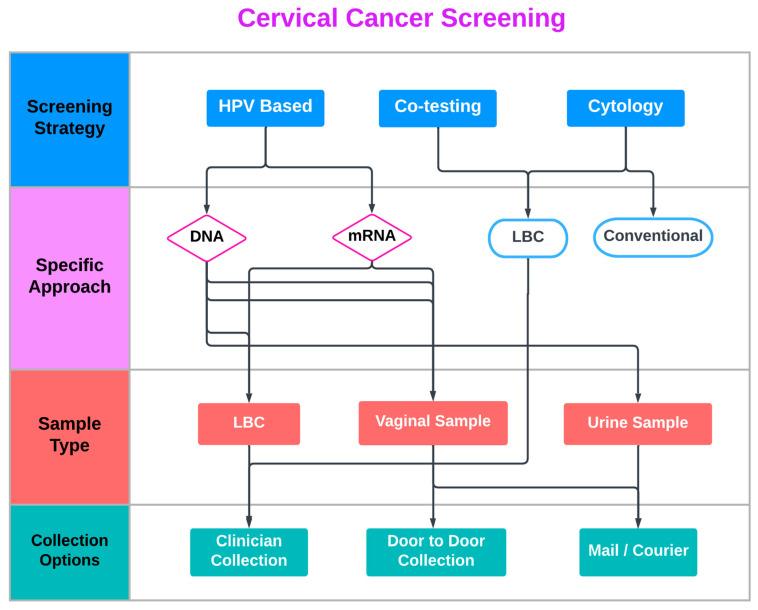
Current Cervical Cancer Screening approaches.

Furthermore, clinician-collected vaginal (or urine) sampling is an emerging alternative option within the cervical screening armamentarium. This strategy represents an interesting approach for women who dislike pelvic exams but otherwise do not feel uncomfortable during office consultation by a physician. Despite the reassuring real-world data so far, the actual proportion of self-samples with inadequate results is very low, and concerns regarding the sampling procedure and the adequacy of the collected specimen are commonly reported by participants in SS studies [26]. Lack of confidence regarding women’s ability to complete the SS procedure correctly is reported in all studies assessing SS acceptability in which women preferred clinician-collected sampling [15]. Interestingly, as the triage of positive SS is currently office based, this particular cluster of patients, acquainted with the presence of a clinician and clinic-based appointments, is less likely to default in case of abnormal (positive) results (Figure 1). 

### 2.7. Guidelines and Endorsements

A constellation of co-factors (the pressure for optimized cost-effectiveness in health services planning, the urge for compliance with the 2020 WHO CC elimination goals and the positive feedback from early SS adopters) has lately culminated in an overtly positive endorsement of SS cervical screening strategies by several scientific bodies and societies, besides the WHO itself [10]. Notably, at the time of this writing, SS does not represent an FDA-approved strategy.

In pragmatic implementation terms, a recent review illustrated that among the 48 countries with HPV-based programs, 17 reported having introduced SS in their national programs or guidelines, either for under-screened populations in eight countries or as the primary screening option for all women in nine countries. Most of these countries have simultaneously introduced the use of HPV-based screening and SS procedures. The majority of countries recommending HPV-based screening are located in Europe and the Americas [47] (Table 1).

### 2.8. Self-Sampling–A Game-Changing Multipotent Liquid Biopsy

Even following the refinement and optimization of platforms and techniques, it is doubtful if self-collected cervicovaginal samples will ever fulfill the high-quality cellularity standards required for morphological review [7]. However, it is clearly evident that SS material represents a unique vehicle where several HPV-related biomarkers and emerging molecular platforms can be assessed and validated [48,49,50,51]. Besides HPV DNA and mRNA studies, this has been so far clearly illustrated in research on viral and host gene methylation [52]. Furthermore, Next Generation Sequencing (NGS), several human microRNAs (miRNAs) and proteins, such as SCCAg, MCSF, and VEGF, as well as other emerging biomarkers besides selected gene polymorphisms, have also shown promising results [7,8,53,54]. Therefore, SS is anticipated to reform the paradigm in cervical screening, underpinning systemic changes, internal upgrades and robust quality assurance. 

## 3. Discussion

It is evident that several gaps in knowledge and uncertainties still exist in SS implementation, mainly in establishing cost-effectiveness and defining ideal triage strategies. Furthermore, there is currently uncertainty about the capacity of HPV testing on self-collected samples to detect the growing threat of cervical adenocarcinoma [34]. The limited data on the influence of SS on CC mortality rates parallels uncertainties on its long-term negative predictive value [34]. In both constrained and affluent environments, stakeholders and policymakers will have to carefully consider several setting-specific factors to fine-tune for successful SS implementation.

Among other authors, Poljak et al. remark that the SARS-CoV-2 pandemic has led to an unprecedented demand for novel molecular tests and platforms [7,55]. However, the global demand for SARS-CoV-2 testing, in particular, is competing with HPV testing combined with a lack of staff, resulting in challenges for current laboratory services and for settings keen to roll out HPV-based screening, further hampered other constraints (self-isolation, quarantine, etc.). Importantly, innovation driven by developments in molecular COVID-19 testing systems may be transferred in the near future toward solutions addressing the shortage of rapid, low-cost HPV testing systems in non-affluent settings. Soon, when the demand for COVID-19 testing is anticipated to decrease eventually, this shift may release both workforce and platform capacity; this can be repurposed following refurbishment and calibration for high-throughput HPV testing [55]. 

Notably, in terms of human resources, Poljak et al. consider that the rapid recruitment and cross-training of staff to support the organization and delivery of COVID-19 testing will ensure a future larger cohort of trained individuals (of varying seniority and experience) will skills highly transferable to general molecular systems, including HPV SS. They conclude that the global health community should invest in opportunities around innovation and capacity to address the CC elimination goals [55].

The SARS-CoV-2 pandemic represented a unique opportunity to reform cervical screening by reinforcing SS. In Sweden, for example, SS targeting long-term non-attenders has been available for a while as a method to increase population coverage but was rarely used. The SARS-CoV-2 pandemic necessitated major emergency changes to longstanding CC preventive strategies; reforms prioritizing organized primary SS for all age groups have been implemented. Therefore, the disruption was used as a milestone opportunity and catalyst to advance the program, with lasting improvements in screening coverage and cost-effectiveness [17]. A similar trend has also been observed in the Netherlands [33].

## 4. Conclusions

Self-sampling (SS) could offer a unique opportunity for mid- and post-pandemic catch-up screening and will play an important role in improving the global coverage of CCS. Indeed, the World Health Organization strongly recommends the use of SS to achieve CC control by 2030 [10,56]. SS should not be only regarded as one of the most convenient strategies for mid- and post-pandemic catch-up screening but a lasting reform with enormous potential [7]. CC has been notoriously known as the disease of inequities; in this context, by lowering the barriers to screening participation SS may ultimately reduce health disparities in women [16,26]. 

With comparable clinical accuracy for hrHPV testing in self-samples as clinician-collected material and flexibility for use both in primary cervical screening as well as triage of abnormal samples, advocacy for incorporating self-sampling into organized screening programs is strong, especially after the disruption due to the SARS-CoV-2 pandemic [5,17,20,21,57,58,59,60]. With an ever-growing number of countries considering self-collection for cervical screening, issues to be determined are how to best offer the service, whom to offer it to, adhering to f-u, triage of HPV-positive cases and how to best prepare the public and health systems for this transition [14,61]

## Figures and Tables

**Table 1:** Current implementation of vaginal self-sampling strategies.

Invitation	SS Collection	Central Lab Sample SS Processing	Triage Using the Same SS Vial	Further Management
Opt-in	Brushes	Expedited shipment in central lab(s)	Methylation	Colposcopy-based
Opt-out (send to all)	Vaginal swabs	Guaranteed stability in extreme temperatures	Dual stain (p16/ki67)?	Colposcopic algorithms tailored for vss depending on the setting (basic, limited, enhanced, maximal)
Digital platforms and social networking	Lavage devices	Resuspension and processing	Other emerging biomarkers	Special populations: expedited treatment integrated within HIV services, etc.
Integration with HIV services	Tampon/patch	Recalibrated HPV DNA PCR assays tailored for SS material		
		Optimized techniques		
		Special reagents		
		New cut-offs		

## Data Availability

The data can be shared up on request.
